# Survival Rates of Dental Implants in Autogenous and Allogeneic Bone Blocks: A Systematic Review

**DOI:** 10.3390/medicina57121388

**Published:** 2021-12-20

**Authors:** Phil Donkiewicz, Korbinian Benz, Anita Kloss-Brandstätter, Jochen Jackowski

**Affiliations:** 1 Department of Oral Surgery and Dental Emergency Care, Faculty of Health, School of Dentistry, Witten/Herdecke University, 58448 Witten, Germany; Korbinian.Benz@uni-wh.de (K.B.); jochen.jackowski@uni-wh.de (J.J.); 2 Faculty of Mathematics and Statistics, Carinthia University of Applied Sciences, Europastrasse 4, 9524 Villach, Austria; A.Kloss-Brandstaetter@fh-kaernten.at

**Keywords:** bone augmentation, implant survival, allogeneic, autogenous, bone block, alveolar ridge, FDBA

## Abstract

*Background and Objectives*: Preliminary studies emphasize the similar performance of autogenous bone blocks (AUBBs) and allogeneic bone blocks (ALBBs) in pre-implant surgery; however, most of these studies include limited subjects or hold a low level of evidence. The purpose of this review is to test the hypothesis of indifferent implant survival rates (ISRs) in AUBB and ALBB and determine the impact of various material-, surgery- and patient-related confounders and predictors. *Materials and Methods*: The national library of medicine (MEDLINE), Excerpta Medica database (EMBASE) and Cochrane Central Register of Controlled Trials (CENTRAL) were screened for studies reporting the ISRs of implants placed in AUBB and ALBB with ≥10 participants followed for ≥12 months from January 1995 to November 2021. The review was conducted in accordance with Preferred Reporting Items for Systematic Reviews and Meta-Analyses (PRISMA) guidelines. The risk of bias was assessed via several scoring tools, dependent on the study design. Means of sub-entities were presented as violin plots. *Results*: An electronic data search resulted in the identification of 9233 articles, of which 100 were included in the quantitative analysis. No significant difference (*p =* 0.54) was found between the ISR of AUBB (96.23 ± 5.27%; range: 75% to 100%; 2195 subjects, 6861 implants) and that of ALBB (97.66 ± 2.68%; range: 90.1% to 100%; 1202 subjects, 3434 implants). The ISR in AUBB was increased in blocks from intraoral as compared to extraoral donor sites (*p =* 0.0003), partially edentulous as compared to totally edentulous (*p =* 0.0002), as well as in patients younger than 45 as compared to those older (*p =* 0.044), cortical as compared to cortico-cancellous blocks (*p =* 0.005) and in delayed implantations within three months as compared to immediate implantations (*p =* 0.018). The ISR of ALBB was significantly increased in processed as compared to fresh-frozen ALBB (*p =* 0.004), but also in horizontal as compared to vertical augmentations (*p =* 0.009). *Conclusions*: The present findings widely emphasize the feasibility of achieving similar ISRs with AUBB and ALBB applied for pre-implant bone grafting. ISRs were negatively affected in sub-entities linked to more extensive augmentation procedures such as bone donor site and dentition status. The inclusion and pooling of literature with a low level of evidence, the absence of randomized controlled clinical trials (RCTs) comparing AUBB and ALBB and the limited count of comparative studies with short follow-ups increases the risk of bias and complicates data interpretation. Consequently, further long-term comparative studies are needed.

## 1. Introduction

Contemporary implant-borne prosthetics represent the benchmark for dental restorations regarding both aesthetics and improvements in the quality of life [1,2]. However, prolonged edentulism, periodontal disease, tumor and cyst resection, trauma and/or infection may cause severe bone loss and render implantation inaccessible [3,4]. Despite several implant-associated innovations, including the development of materials with greater Young’s modulus than conventional titanium [5], implant surface modification [6] or the introduction of narrow and short implants [7], pre-implant bone regeneration is often inevitable, as emphasized by an analysis of 10,158 implants which revealed a bone grafting frequency of 58.2% [8]. Various grafting materials and techniques have been introduced to restore lost bone tissue and facilitate implantation, and former comparative studies have reported similarly favorable outcomes for implants in pristine or augmented bone; however, one study found implant survival rates (ISRs) of 90% for implants in native bone and 79% for implants in grafted bone following ten years of healing [9], whereas others have reported cumulative ISRs of 94.1% for implants in native and 100% for those placed in grafted bone [10].

Grafting materials with the intrinsic capacity of producing new bone tissue via vital osteoblasts are called osteogenic, whereas grafting materials stimulating the differentiation of progenitor cells into osteogenic cells, which is most frequently mediated by growth factors such as bone morphogenetic protein-2, have osteoinductive properties, and materials solely functioning as a scaffold by stabilizing the defect area and providing structural support for new bone formation are termed osteoconductive [11]. Autogenous bone grafts exhibit all three characteristics, whereas most commercially available bone substitute materials are only osteoconductive [12]. The most obvious disadvantage of autogenous bone grafts is the bone harvesting procedure and the associated donor site morbidity, whereby harvesting from extraoral sites, including the iliac crest, is associated with a considerably increased risk of complications and patient burden as compared to bone harvesting from intraoral sites, especially the ramus [13,14,15]. In this context, pain score analysis of patients enrolled in a study comparing bone augmentation carried out with autogenous bone blocks (AUBBs) and allogeneic bone blocks (ALBBs) was in favor of patients who were treated with ALBBs and was concomitant with a higher willingness to undergo the procedure again [16].

Allografts are manufactured from deceased or living human bone donors, which were established to combat the challenges of availability and harvesting of autogenous bone and donor site morbidity [17]. Due to their physicochemical properties, which widely resemble those of autografts, and similar clinical outcomes, allografts have been proposed to represent the best option for autografts [18,19]. Implant dehiscence and minor horizontal bone defects are predictably restorable with various granular bone substitute materials in most instances, whereas the treatment of complex osseous defects spanning multiple teeth or entire jaws requires extensive measures such as titanium meshes or block bone grafts [20]. Regarding the application of solid bone blocks, solely allogeneic bone blocks (ALBBs) have been demonstrated to result in comparably favorable outcomes to autogenous bone blocks (AUBBs), whereas clinical data of xenogeneic or alloplastic block grafts are vastly limited and generally discourage their application [21,22,23]. In a previous review, Nevins et al. reported an ISR of 97.5% for 526 implants followed from 6 to more than 74 months post-loading, irrespective of the application of autogenous or allogeneic bone blocks [24]. Another systematic review by Motamedian et al. found ISRs ranging from 73.8% to 100% for autogenous and 95.3% to 100% for allogeneic bone blocks [25], which is consistent with the findings reported in other reviews [26,27,28].

Most previous reviews solely include a small set of the available literature, do not provide statistical analyses and/or denote a lack of evidence in order to draw conclusions on the performance of AUBBs and ALBBs in pre-implant augmentative surgery; therefore, we chose to conduct a comprehensive analysis of the ISR subjected to the effect of confounders. With the establishment of sub-entities, the impact of bone donor site, type of allograft, the cortical and cancellous composition, inlay and onlay bone grafting, applications of barrier membranes and relining materials, horizontal and vertical bone defects, dental status and age of the patients, graft location, graft consolidation times and follow-up duration were considered in the statistical analysis. Through this approach, combined with the inclusion of the broad scope of available information, we sought to further the understanding on the feasibilities and limitations of autogenous and allogeneic bone blocks in pre-implant augmentative surgery.

## 2. Materials and Methods

This systematic review was carried out in accordance with the PRISMA (Preferred Reporting Items for Systematic Reviews and Meta-Analyses) guidelines with the purpose of evaluating the ISRs of implants in autogenous and allogeneic bone blocks used for pre-implant surgery [29]. Registration at PROSPERO was no longer feasible due to changes in guidelines during our review process; therefore, we researched their database, but could not identify protocols resembling ours. We used the PICO (Patient—Intervention—Comparison—Outcome) framework to elaborate the following question: Does a significant difference between the survival rate of implants placed into autogenous and allogeneic bone blocks applied for pre-implant augmentations exist, and which impacts do various material-, surgical- and patient-related confounders and predictors elicit? The ISRs of systemically healthy patients who underwent bone regeneration with AUBBs were compared to the ISRs of those who received ALBBs for bone regeneration.

### 2.1. Search Strategy

The National Library of Medicine (MEDLINE-PubMed), the Cochrane Central Register of Controlled Trials (CENTRAL) and the EMBASE (Excerpta Medica database) databases were searched from 1 January 1995 to 7 November 2021 using the following terms: “implant survival”, “bone allograft”, “alveolar bone grafting”, “autogenous bone graft”, “bone augmentation”, and the MeSH (Medical Subject Headings) term (“Bone Transplantation” OR “Alveolar Bone Grafting”[MeSH] OR “Alveolar Ridge Augmentation”[MeSH]) AND (“Dental Implants”[MeSH] OR “Dental Implantation”[MeSH]) AND (“Graft resorption”[MeSH] OR “Survival Rate”[MeSH]) AND (“Transplantation, Autogenous”[MeSH] OR “Transplantation, Homologous”[MeSH]). If applicable, the following set of filters was used: Clinical Study, Clinical Trial, Comparative Study, Controlled Clinical Trial, Multicentre Study, Observational Study; Dental Journals; Human applications. This literature search was supplemented by a hand search of the following journals: *Clinical Implant Dentistry and Related Research*, *Clinical Oral Implants Research*, *Implant Dentistry,* the *Journal of Clinical Periodontology*, the *Journal of Oral and Maxillofacial Surgery*, the *Journal of Periodontology*, the *Journal of Oral Implantology*, and the *Journal of Oral and Maxillofacial Implants*, and cross-checking the reference lists of selected studies and review articles to identify additional publications eligible for inclusion.

### 2.2. Eligibility Criteria

The inclusion criteria were as follows:

- Studies on human subjects;
- Publications in English;
- Randomized controlled clinical trials, controlled clinical trials, prospective and retrospective clinical studies reporting the application of autogenous and allogeneic bone blocks;
- Studies describing the implant survival rate in at least 10 study subjects;
- Studies following implants for at least 12 months.

The exclusion criteria were as follows:

- Animal studies;
- Publications of identical data in follow-up studies;
- Previous data of identical cohorts in older publications;
- Non-English publications;
- Studies on patients suffering from cancer, metabolic, immunologic or other systemic diseases;
- Studies not reporting absolute implant survival rates.

### 2.3. Screening Process and Study Selection

Abstracts of articles derived from the literature search were screened for eligibility criteria, and if these were met, the full texts were acquired for in-depth analyses. The first reviewer (P.D.) conducted the primary screening and preselection of studies presumably meeting the inclusion criteria. In cases of missing useful elements in the title or abstract, studies were processed for full-text analysis. Following the initial screening process, the other two reviewers (K.B. and J.J.) independently evaluated the preselected studies. All studies that met the inclusion criteria were processed for validity assessments to identify ineligible records and duplicates. The remaining studies were forwarded for quality assessment and data extraction.

### 2.4. Data Collection and Assessment

Data were extracted by the first reviewer (P.D.) and controlled independently by two other investigators (K.B. and J.J.). Heterogeneity between the selected studies and the included study populations was assessed by recording the following items:

- Study design;
- Graft type/donor site;
- Cortical/cancellous composition;
- Inlay/onlay grafting;
- Materials for resorption protection;
- Horizontal/vertical bone defect;
- Dentition status;
- Treatment in the mandible/maxilla;
- Mean age of patients;
- Mean time to implantation;
- Duration of follow-up.

The outcome of interest was the ISR, which was assessed from implants failing within a follow-up period of at least 12 months post-insertion. If the mean follow-up was not available, the shortest time span reported was considered for all implants. Implant success criteria were heterogeneously defined among studies and less frequently reported; therefore, no analysis of these was conducted. The effect of confounders, namely, graft type (GT; CV, calvaria; CH, chin; IC, iliac crest; RA, ramus, FFBA, fresh-frozen bone allograft; PBA, [wet chemico-physically] processed bone allograft; DFDBA, demineralized freeze-dried bone allograft; FDBA, freeze-dried bone allograft; SDBA, solvent-dehydrated bone allograft), the cortical and cancellous composition of the graft material (Co/Ca/CoCa), inlay and onlay block grafting (in/on), application and type of resorption barriers (RB; PTFE, polytetrafluoroethylene membrane; RCM, resorbable collagen membrane; RL, relining of the graft with granular material; Ti-MH, titanium mesh; TS, tenting screw), horizontal and vertical bone augmentations/defect type (DT; H/HV), dentition status (DS; PE, partially edentulous; TE, totally edentulous), grafting location (GL; Mn, mandible; Mx, maxilla) as well as age (<45 years, young; ≥45 years, old), immediate, early and late implant insertion after grafting (i.i.; e.i., <6 months; l.i, ≥6 months), and short and long follow-up times (SFUP/LFUP) was separately analyzed when at least five studies were eligible for inclusion.

**Figure 1:**
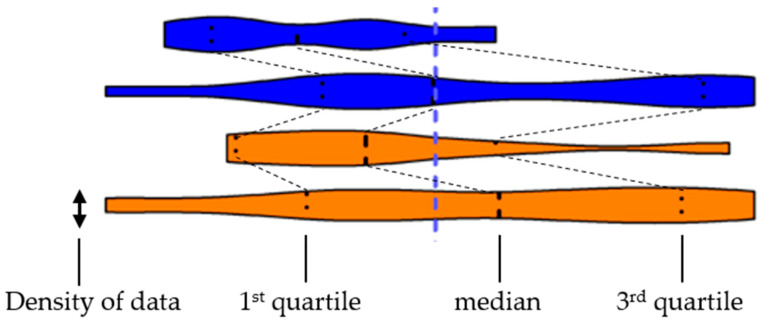
Demonstration of a violin plot and the depicted data. Three lines within the plot show the 1st and 3rd quartile and the median of the dataset, whereas the width of the violin body indicates the density of data along the x-axis. The edges of the violins represent the minimum and maximum values of the dataset.

Differently from a former systematic review [30], statistics were not calculated with weighted means but with means reported for individual study groups. We assumed that withdrawing the impact of weighting would lower the risk of bias caused by the surgeon, because monocentric studies with large patient collectives may determine a large proportion of the overall value with this method. Results were illustrated as truncated violin plots, because these depict a comprehensive overview on the distribution of individual data by the shape of the violin body, which alleviates the identification of similar or equal datasets included for establishing distinct subgroups (Figure 1).

### 2.5. Quality Assessment and Risk of Bias

Studies with different levels of evidence and various study designs were included in this analysis; therefore, a uniform assessment tool based on the National Institute of Health (NIH) quality assessment tool for case series studies was employed for the assessment of all studies. To improve quality assessments throughout different types of studies, the catalogue of nine items was adapted into three, namely, “study design”, “presence of a registered study protocol” and “sample size”. The overall number of points assessed for each study was divided by the number of applied items to calculate the quality score. Studies with 80% and above were rated as “good”, those with 60–79% were rated as “fair” and studies with a score of 59% and below were rated “poor”.

Comparative studies were additionally evaluated by the Newcastle–Ottawa Scale (NOS) [31], which was developed to assess the quality of cohort-studies; however, it widely overlaps with the items of the modified NIH (mNIH) scoring tool. The Jadad scale [32], which comprises seven items specifically addressing randomization, blinding and follow-up, was also implemented as a second scoring tool for RCTs. A detailed overview of the applied quality assessment tools and scoring of all studies included is provided in Appendices C–H. Quality assessment was conducted by the first reviewer (P.D.) and controlled by two independent reviewers (J.J. and K.B.).

### 2.6. Data Analysis

Data analysis was conducted by the first reviewer (P.D.) and subsequently controlled by an additional reviewer and statistics professor (A.K.-B.). The Shapiro–Wilk test (SWT) was applied to check for data distribution; for singular comparisons of parametric datasets, the unpaired Student’s t-test was conducted, whereas the Mann–Whitney U test (MWU) was applied for nonparametric data. The overall mean calculated for autogenous and allogeneic bone blocks was compared to that of each respective subgroup. Depending on the distribution of data, either an ANOVA (analysis of variance) in combination with an uncorrected Fisher’s LSD test or the Kruskal–Wallis test together with an uncorrected Dunn’s test was applied for the comparison of the overall ISR with the respective subgroups to test the hypothesis of different ISRs in AUBBs as compared to the individual sub-entities. We chose uncorrected tests because we considered each comparison to be independent; additionally, because the α correction is calculated by the number of comparisons being carried out, an uneven number of established sub-entities would impact significances and, consequently, cause bias. Corresponding subgroups established for AUBB and ALBB were subjected to intergroup comparisons, whereas distinct subgroups of AUBB and ALBB were compared in intragroup analysis.

In cases of three or more interdependent values within one study item, e.g., cancellous, cortical, and cortico-cancellous AUBB, multiple comparisons with correction were carried out so that analysis of variance (ANOVA) with Bonferroni correction could be applied for parametric data and the Kruskal–Wallis test with Dunn’s correction (KWT) could be used for non-parametric data. The overall ISR of AUBB was compared with the overall ISR of ALBB, but also to that calculated for FFBA and PBA, because these represent the two sub-entities solely related to the material quality of ALBB; similarly, the overall ISR obtained for ALBB was compared to that of AUBB harvested from intra- and extraoral donor sites, whereby statistics were again calculated via corrected ANOVA or KWT. Significance was set at *p* < 0.05. GraphPad Prism 9 software (GraphPad Software, Inc., San Diego, CA, USA) was used for statistical analysis in this systematic review.

## 3. Results

### 3.1. Selected Studies

**Figure 2:**
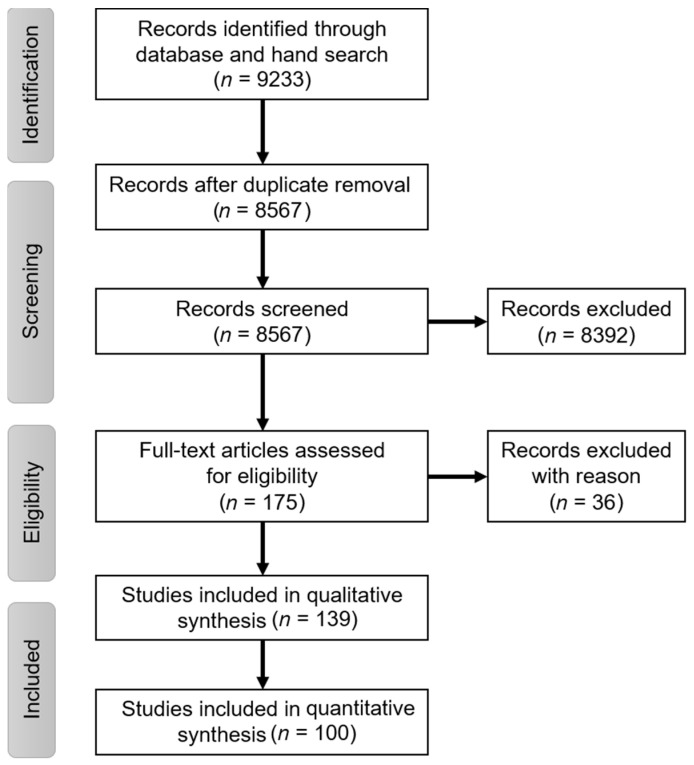
PRISMA flowchart diagram of the study identification and selection process.

The electronic database and hand research resulted in the identification of 9233 articles, of which 8567 remained following the removal of duplicates. After screening titles and abstracts, 8392 studies were excluded and 175 studies were processed for full-text analysis, which resulted in the exclusion of another 36 studies; as a result, 139 studies remained for the qualitative synthesis in this review. A total of 100 studies, of which 71, 23 and 6 reported results associated with autogenous, allogeneic and both type of bone blocks, respectively, were included into the quantitative synthesis. A table of excluded studies with the reasons for exclusion is provided in Appendix B. There were 14 and 24 RCTs and controlled cohort-studies associated with AUBB, respectively, whereas 2 RCTs and 7 cohort-studies associated with ALBB were identified. A PRISMA flowchart diagram of the screening process is depicted below (Figure 2). Studies associated with AUBB were identified from the year 1996 onwards, whereas the first studies of ALBB suitable for inclusion were published one decade later (Appendix A).

### 3.2. Study Quality

The calculated mean mNIH, NOS and Jadad scores of studies associated with AUBB were 70%, 83% and 61%, respectively, whereas those scores for studies regarding ALBB were 80%, 100% and 70%, respectively. The mNIH and NOS scores of studies comparing AUBB and ALBB were 0.69% and 92% (Appendix H). Overall, the rating of all studies associated with AUBB was “fair” when assessed by the mNIH score and “good” when assessed via the NOS score, whereas both mNIH and NOS for studies associated with ALBB were “good”. According to the mNIH score, 17 studies (22%) on AUBB were rated “poor”, 48 (62%) were rated “fair” and 12 were rated “good”. As for ALBB, 2 studies (7%) were rated “poor”, 19 (65%) were rated “fair” and 8 (28%) were rated “good”. Studies reporting outcomes of AUBB lost most points due to the absence of or failure to mention a registered study protocol (66%), followed by the missing consecutiveness of treated subjects (48%), weak sample size (44%) and a retrospective study design (44%). A total of 30 (39%) studies conducted with AUBB had a follow-up exceeding 36 months, 14 (18%) studies ranged between 25 and 36 months and 33 (43%) had a follow-up of 12 to 24 months (Appendix F). As for ALBB, most points were deducted because of a lack of information on whether study subjects were treated consecutively (66%) followed by a retrospective study design (38%), a follow-up of less than 24 months (34%), sample sizes of fewer than 20 subjects (34%) and absence of a registered study protocol (31%; Appendix G). Regarding the evaluation via NOS, 72% of studies on AUBB lost one point because they did not control for an additional factor or outcome, and 50% lost one point because the outcome of interest was already present at the start. All RCTs included in this analysis were labelled as such; hence, they were all awarded one point for randomization according to the Jadad scale. However, 71% and 50% of the included RCTs on AUBB and ALBB, respectively, did not mention a blinding method. Additionally, 43% and 50% of studies either failed to mention blinding or blinding was not conducted (Appendix F and Appendix G).

### 3.3. Implant Survival Rate

**Figure 3:**
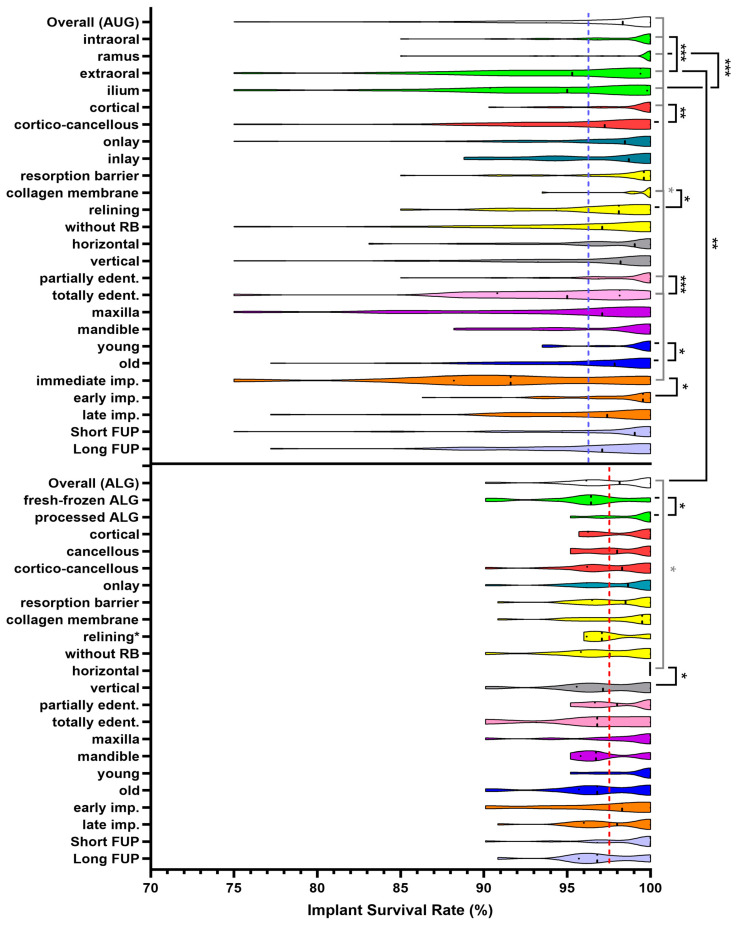
Implant survival rate of autogenous and allogeneic bone blocks calculated from mean values of study groups illustrated as violin plots. Dots within the violin body demonstrate the 1st and 3rd quartiles, whereas the dashed line indicates the median. Blue dashed lines show means of AUBB; red dashed lines show those of ALBB. Black bars represent comparisons via Mann–Whitney U test; grey bars label the comparison of the overall ISR with the sub-entities via the uncorrected Dunn’s test (*p* < 0.05 */0.005 **/0.0005 ***); AUG: autogenous bone graft; RB: resorption barrier; FUP: follow-up; ALG: allogeneic bone graft.

A total of 77 studies reported a mean ISR of 96.23 ± 5.19% (range: 75% to 100%) associated with 6861 implants placed within 2397 AUBBs applied in 2195 patients (1.1 grafts/patient; 3.1 implants/patient; 2.9 implants/graft) with a mean follow-up of 38.3 ± 36.7 (range: 12 to 144) months, whereas 29 studies reported the survival rate of 3434 implants placed in 1384 ALBBs used for bone regeneration in 1202 subjects (1.2 grafts/patient; 2.9 implants/patient; 2.5 implants/graft) with a mean follow-up of 25.1 ± 10 (range: 12 to 60) months, which was 97.74 ± 2.67% (range: 90.1% to 100%; Table 1, Table 2 and Table 3). Statistical analysis indicated no significant difference between the overall ISRs of AUBB and ALBB (*p* = 0.54; Table 4). The mean graft consolidation time until implantation was 5.00 ± 1.36 months, and the mean age of subjects was 50.06 ± 8.31 years for studies reporting ISR in AUBB and 5.93 ± 1.01 months and 47.97 ± 11.64 years for studies reporting ISR associated with ALBB. Due to the larger range of ISRs, the violins of groups established for AUBB were elongated when compared to ALBB. The violin plot analysis demonstrated that apart from one sub-entity established for AUBB, namely, immediate implant placement, the median ISRs of all established sub-entities were above 95% (Figure 3).

The Shapiro–Wilk test indicated a nonparametric distribution of the dataset (*p* < 0.0001), whereas the Kruskal–Wallis test was applied to test for potential differences regarding the overall ISR of AUBB and the individual sub-entities and indicated a highly significant difference between the overall ISR of AUBB and all established sub-entities (*p <* 0.0001; Table 4). The subsequent multiple comparison analysis via the uncorrected Dunn’s test indicated that the ISRs significantly increased in intraoral (*p =* 0.045), ramus (*p =* 0.011) and cortical (*p =* 0.048) AUBBs as well as in AUBBs covered with a barrier membrane (*p =* 0.021) and AUBBs applied in partially edentulous patients (*p =* 0.049) when compared to the overall ISR of AUBB. Additionally, the ISRs in extraorally harvested (*p =* 0.023) and iliac crest (*p =* 0.035) grafts were significantly lower as compared to the overall ISR of AUBB, similarly to the ISRs in totally edentulous patients (*p =* 0.021) and survival of immediate implants (*p =* 0.023). The Kruskal–Wallis test indicated no statistically significant difference when comparing the overall ISR of implants in ALBB with all established sub-entities (*p =* 0.342), although a significantly increased ISR for horizontal bone augmentations (*p =* 0.022) as compared to the overall ISR of ALBB was detected by multiple comparison analysis via the uncorrected Dunn’s test (Table 4).

The Mann–Whitney U test, which was applied to detect for significance between the two study entities, indicated that the overall ISR of implants in ALBB was significantly higher than that of implants inserted into AUBB harvested from extraoral (*p =* 0.002), but not from intraoral (*p =* 0.94; Table 5) donor sites. Likewise, the ISR in grafts harvested from intraoral sites was significantly increased as compared to the ISR in grafts from extraoral donor sites (*p =* 0.0003), similarly to ISR in cortical as compared to cortico-cancellous (*p =* 0.005) AUBB. Additionally, the ISR in totally edentulous patients treated with AUBB was significantly lower than that of partially edentulous patients (*p =* 0.0002) receiving AUBB for pre-implant bone regeneration, but also for blocks covered with relining materials as compared to AUBB solely covered with membranes (*p =* 0.019) in patients older than 45 years as compared to younger patients (*p =* 0.044), and in early implantations within five months of healing as compared to immediate implantation procedures (*p =* 0.018). Regarding ALBB, the Mann–Whitney U test demonstrated a significantly increased ISR in processed ALBB as compared to fresh-frozen ALBB (*p =* 0.004) and for implants inserted in ALBB used for horizontal as compared to vertical bone augmentation procedures (*p =* 0.009; Table 5; Figure 3).

## 4. Discussion

### 4.1. Study Selection and Quality Assessment

Although a slightly higher quality was assessed for studies reporting the results of ALBB as compared to AUBB with each of the three evaluation tools applied, the variations were negligible. Studies assessed via the NOS exhibited an increased quality, whereas studies assessed via the Jadad scale had a decreased quality when compared with their respective mNIH scores. Considering that the most frequent items responsible for quality degradation were the absence of a registered study protocol, missing consecutiveness, and weak sample sizes, which are all items not being analyzed with the NOS, it is obvious why studies exhibited a higher score with this tool (Appendix C and Appendix D). As in the Jadad scale points, are deducted in cases of missing items of interest, the absence of mentioning blinding, which applied to the majority of included RCTs, and the concomitant fail to mention the blinding method, which resulted in two out of five points not being awarded, and additionally, one point being deducted. Consequently, even if all other items were fulfilled by the respective study, the overall score would only be 40%. Therefore, it is obvious why the mean Jadad score of RCTs was lower as compared with their corresponding mean mNIH scores. Studies on ISR in AUBB were identified one decade earlier than studies reporting ISR in ALBB, which is the main reason for the increased count of studies reporting results about AUBB (Appendix A).

Only 6 [16,126,127,128,129,130] out of the 100 included studies were direct comparisons of AUBB and ALBB; no RCTs were among these studies, and none was carried out in a split-mouth fashion (Appendix E). Additionally, the fact that the preliminary data selection was exclusively conducted by the primary investigator increased the risk of selection bias. Due to these shortcomings and the heterogeneity among the identified studies, which poses a known issue related to this subject and has previously been reported by others [131], we decided to refrain from the conduction of a meta-analysis but performed simple statistical tests with the values obtained for the established sub-entities similar, to a previous systematic review [30]. The assessed overall mNIH scores were 70% (fair) for AUBB and 80% (good) for ALBB and indicated a moderate risk of bias among the selected studies. In this context, it should be noted that all included studies were published in peer-reviewed medical journals; thus, all data have previously been controlled by third parties. The compromise for the generation of this large dataset was the inclusion and pooling of results from studies with varying study designs, defect morphologies and locations, graft types and compositions as well as follow-up durations; therefore, the results presented here should be further validated by randomized and controlled clinical trials.

### 4.2. Implant Survival Rate

The survival of implants within grafting materials is probably the most crucial analysis, because the main purpose of bone grafting procedures is dental rehabilitation, which should last decades, and ideally, a lifetime. As for AUBB, two studies reported implant survival rates (ISRs) below 80% for cortico-cancellous iliac grafts with simultaneous [34] and delayed implants [74] in the maxilla, and both studies included totally edentulous patients. Both authors reasoned the loss of implants with marginal bone resorption, which was demonstrated to be increased within the first three years. Astrand et al. concluded that proceeding with the resorption of AUBB, especially with grafts exhibiting large amounts of cortical bone, which is impervious to vascular infiltration, is one major challenge for the predictability and the long-term success of implants in AUBB [34]. In this context, Molly et al. reported a significantly decreased marginal bone resorption of less than 1 mm associated with implants inserted into bone which was regenerated via titanium foil without a grafting material when compared to the marginal bone loss of implants placed in cortico-cancellous AUBB, which was 2.7 mm after 20 years, and hence demonstrated ongoing block resorption [74].

A cohort study comparing the survival of implants placed in jaws augmented with cortico-cancellous iliac blocks covered by an RCM (resorbable collagen membrane) alone or by an RCM combined with deproteinized bovine bone mineral (DBBM), reported ISRs of 99.2% and 98.8%, respectively, but due to the significantly lower resorption the authors observed in the DBBM group, they reasoned that graft covering with non-resorbable materials might be beneficial regarding long-term outcomes, whereby follow-up in this study did not exceed 24 months; thus, this was insufficient for a definitive conclusion [101]. A previous study of the same group reporting a 91.5% 5-year implant survival rate with uncovered iliac grafts which emphasized their conclusion [102]. Additionally, Tunkel and De Stavola made similar observations when covering augmentation sites restored via the shell technique with DBBM at the time of implant insertion, which they established as the “delayed relining technique” [131]. In contrast to these findings, an RCT reported 2-year ISRs of 100% for a cohort receiving uncovered AUBB, but also AUBB covered with DBBM and an RCM, whereby the authors described higher complication rates associated with the application of barrier materials [48]. Two RCTs reported slightly reduced ISRs within cortico-cancellous chinbone [71] and ramus [60] grafts covered with DBBM and an RCM as compared to uncovered grafts, whereby the authors of both studies stated that all applied augmentation techniques resulted in comparably favorable outcomes. In our analysis, we also found an increased implant failure rate for implants inserted into relined AUBB as compared to blocks covered only by an RCM (*p =* 0.021); however, the ISR of uncovered AUBB was lower than that of grafts covered by membranes and/or granular materials, whereby the difference was not statistically significant (*p =* 0.071). One important issue might be the difference between graft relining and graft overcontouring. Relining is based on a thin layer of volume-stable grafting material, whereas overcontouring marks the extending of the graft beyond the bony envelope, which has been demonstrated to be accompanied by increased complication rates [132]. Further controlled cohort studies with long-term follow-up should investigate the impact of graft relining.

Regarding the relevance of the bone donor site and the patient’s dentition, Raghoebar et al. reported an ISR of 100% for partially edentulous patients receiving cortico-cancellous AUBB harvested from intraoral sites, whereas an ISR of 95.6% was found for totally edentulous patients treated with bone blocks from the iliac crest [82,83]. These findings strongly correlate with the results from our statistical analysis, which demonstrated significantly increased ISRs in grafts harvested from intraoral as compared to extraoral donor sites, and likewise, in grafts harvested from the ramus as compared to those harvested from the iliac crest. The significantly increased ISR calculated for cortical as compared to cortico-cancellous AUBB is strongly linked to these findings because, other than cortico-cancellous iliac grafts, ramus grafts are mostly monocortical. Totally edentulous patients are more often subjected to bone augmentation with grafts harvested from extraoral donor sites than partially edentulous patients; therefore, ISRs calculated for implants in partially edentulous patients were significantly increased. In this context, the significantly lower ISRs of implants in AUBB determined for patients older than 45 as compared to those of younger patients may, on one hand, result from age-related causes, but also from the extensive crossover of this dataset with that of the totally edentulous group. Statistical analysis indicated a significantly lower ISR for immediate implants as compared to delayed implants placed within five months of healing, but not as compared to implants inserted following six or more months of graft consolidation, which makes sense considering that extraoral bone grafts have prolonged healing times and, hence, are stronger represented in the late implantation group, whereby some authors reported similar ISRs for immediate and delayed implants [77,78,80].

Implant survival rates in all studies associated with the application of ALBB were above 90%, whereby the lowest survival, which mainly resulted from insufficient primary implant stability, was reported for implants inserted into FFBA onlay blocks applied without resorption barriers [115]. The second lowest ISRs of 94.03% were also reported for cortico-cancellous FFBA blocks. The inclusion of full-arch reconstructions, and thus, complex bone augmentations with extensive bone volumes, for which statistical analyses of AUBBs already indicated lower ISRs as compared to segmental reconstructions in partially edentulous patients, is one putative reason for the rather low ISRs reported in this study [111]. Studies reporting results associated with totally edentulous patients were exclusively conducted with FFBA, and 90% of these included vertical defects, for which our analysis indicated a significantly lower ISR as compared to implants inserted in ALBB applied for horizontal bone defects. This, in turn, provides an explanation for the significantly increased ISR found for processed bone allografts (PBAs) as compared to FFBA blocks. In two independent studies by the same authors, ISRs of 98.3% and 96.3% were reported for cortico-cancellous and cortical FFBA block grafts, respectively [107,108], whereas other authors reported 2-year ISRs of 100% for cortico-cancellous and cortical FDBA blocks alike [125], which further emphasizes the results of the statistical analysis in this review (Table 5). Nissan and Chaushu also analyzed survival rates of up to three years for implants inserted into cancellous FDBA blocks, which ranged from 95.2% for restored congenitally missing teeth in the maxilla, whereby only one immediately loaded implant failed [120], to 100% for implants inserted into the augmented anterior mandible [117].

Regarding comparative cohort studies, Al-Abedalla et al. reported similar ISRs for implants in native bone, AUBB and ALBB; additionally, the authors found striking similarities in the tissue composition in histologic specimens of pristine bone and specimens recovered from the augmented area [126]. Chiapasco et al. found 100% 2-year ISRs for cortico-cancellous AUBBs applied for full-arch reconstructions in the maxilla, whereas the ISR of the cohort treated with cortico-cancellous FFBA blocks was 90.1% due to increased complications, graft exposure and uncontrollable resorption. The authors concluded that FFBA does not represent a safe alternative to AUBB [128], which they substantiated by a further study with corresponding results [128]. An implant survival rate of 100% was reported for implants inserted into both cortical AUBB and cortico-cancellous FFBA blocks applied for horizontal bone augmentations in two separate studies by the same authors [34,103], which emphasizes the feasibility of achieving similar results with AUBB and ALBB following the regeneration of horizontal bone defects. An ISR of 100% for both cohorts was reported by all comparative studies on AUBB and processed allogeneic bone blocks. Kloss et al. compared the 1-year ISR of implants in cancellous FDBA blocks with that of AUBB harvested from the ramus for horizontal augmentations in single-tooth defects [129], whereas Park et al. reported 2-year ISR following vertical augmentations with cortical AUBB and cortico-cancellous FDBA [130]. Schlee et al. reported a 2-year ISR of 100% for implants inserted into either AUBB harvested from the ramus or pre-milled bone blocks made of cancellous solvent-dehydrated bone allograft used for the augmentation of three-dimensional bone defects [16].

The six comparative cohort studies [16,126,127,128,129,130] presumably represent the most valid source of contemporary information on the clinical performance of AUBB and ALBB; however, none of these was an RCT and three studies had a retrospective study design, which increases the risk of bias. The longest follow-up for allogeneic bone blocks in those comparative studies was limited to 32.9 months [16], i.e., less than three years. Additionally, the mean follow-up for studies reporting ISRs associated with ALBBs was only 25.1 ± 10 months. Regarding AUBB, the longest follow-up in comparative studies was 59.9 months [130]; additionally, the mean follow-up duration of 38.3 ± 36.7 months of studies reporting ISRs in AUBB notably exceeded that of ALBB. The scarce number of studies with long-term follow-up data represents another shortcoming in the interpretation and analysis of the present data, because only 1–2% of implants fail within the first months, whereas 5% of implant failures occur several years after successful osseointegration of the implant, mainly due to peri-implant disease [133]. Although some authors reported increased complication rates with allogeneic bone grafts, especially in vertical augmentations [134], the majority of clinical studies and previous reviews on this matter emphasize that ALBBs represent an adequate alternative to AUBBs, especially concerning processed bone allografts and horizontal bone regeneration procedures. An extensive analysis of complications occurring in 137 ALBBs by Chaushu et al. found 8% of total graft failures due to “membrane exposure”, “incision line opening”, “soft tissue perforations” and “recipient site infections” [135]. These failure items emphasize the most critical factor for success irrespective of the applied graft: proper soft tissue covering of the graft. One factor clearly favoring the application of ALBB over AUBB is the lower overall patient burden, because no bone harvesting is required. Several studies have reported complications associated with bone harvesting and a low willingness of patients to repeat the same procedure [15,136]. Although the complications are limited to temporary but also permanent nerve damages when bone is harvested from intraoral sites in most instances, more severe complications may occur with extraoral bone harvesting [137,138,139].

## 5. Conclusions

The present findings widely emphasize similar survival rates of implants placed in either autogenous or allogeneic bone blocks. The respective overall ISRs of 96.23 ± 5.27% and 97.66 ± 2.68% determined for AUBB and ALBB indicate high predictability with both these materials in pre-implant augmentative surgery, especially when considering that bone blocks are usually applied for the regeneration of large osseous defects. Ongoing marginal bone loss, which mainly affected extraoral bone grafts in totally edentulous patients older than 45 years, is one challenge associated with AUBB. In this context, limited evidence suggests that the relining of grafts with volume stable materials such as bovine bone mineral may be beneficial for containing graft resorption. Fresh-frozen ALBB resulted in rather unfavorable outcomes in comparative cohort studies comparing them with AUBB, especially regarding vertical augmentations and full-arch reconstructions, whereas the results of processed ALBB were equally comparable with those of AUBB. However, no studies demonstrating the feasibility of full-arch reconstructions with processed ALBB were identified in the literature, and the pooling of studies with different design and study populations, as well as the absence of high-quality comparative studies with long-term follow-up durations, further limit the analysis and interpretation of the present data. Consequently, these preliminary findings should be validated by further comparative long-term studies with high levels of evidence.

## Figures and Tables

**Table 1:** Survival rates of implants placed in studies conducted with autogenous bone grafts. SD, study design; Co/Ca, cortico-cancellous; in/on, inlay/onlay blocks; RB, resorption barrier; DT, defect type; DS, dentition status; GL, grafting location; *n*(P), count of patients; *n*(G), count of grafts; *n*(I), count of implants; TtI, time to implantation; F-Up, follow-up; ISR (%), implant survival rate (%); RCT, randomized controlled trial; PS, prospective study; RS, retrospective study; RA, ramus; CH, chin; IC, iliac crest; CV, calvaria; RCM, resorbable collagen membrane; DBBM, demineralized bovine bone mineral; HA, hydroxyapatite; PRGF, plasma rich in growth factors; H, horizontal; V, vertical; PE, partially edentulous; TE, totally edentulous; Mx, maxilla; Mn, mandible; GT, graft type; FFBA, fresh-frozen bone allograft; FDBA, freeze-dried BA; DFDBA, demineralized FDBA; SDBA, solvent-dehydrated BA. PBA, processed bone allograft.

Author	SD	GT	Co/Ca	in/on	RB	DT	DS	GL	*n* (P)	*n* (G)	*n* (I)	Age	TtI	F-Up	ISR (%)
Acocella et al., 2010 [33]	PS	RA	Co	on	-	H	PE	Mx	15	15	30	41.0	6.0	12.0	100
Altiparmak et al., 2020 [34]	RS	io	CoCa	on	DBBM/PRF	HV	PE	v	53	53	77	56.3	6	60	96.3
Astrand et al., 1996 [35]	RS	IC	CoCa	on	-	HV	TE	Mx	17	17	92	-	DI	12.0	75
Bartols et al., 2018 [36]	RCT	RA	Co	on	RCM	H	PTE	Mx	15	15	15	-	4.0	12.0	100
Bell et al., 2002 [37]	RS	IC	CoCa	on	-	HV	TE	Mn	14	14	70	59.0	5.0	24.0	100
Bienz et al., 2020 [38]	RCT	io	CoCa	on	RCM/DBBM	HV	PE	-	12	12	20	47.5	4	36	100
Bormann et al., 2010 [39]	PS	RA	Co	in	RCM	HV	PE	Mn	13	22	41	48.0	3.0	12.0	100
Bormann et al., 2011 [40]	RS	RA	Co	in	RCM	HV	PE	Mn	27	40	88	58.7	3.0	17.6	100
Boronat et al., 2010 [41]	RS	io	-	on	-	H	PE	-	37	39	73	-	DI	12.0	95.9
Boven et al., 2014 [42]	RS	IC	CoCa	on	-	V	TE	Mn	40	40	80	61.0	4.0	60.0	98.7
Buser et al., 2002 [43]	PS	CH	-	on	ePTFE	H	PE	-	40	40	61	-	7.5	60.0	100
Chappuis et al., 2016 [44]	PS	io	-	on	RCM/DBBM	H	PE	-	38	38	52	45	6.3	133	98.1
Chiapasco et al., 1999 [45]	PS	io	-	on	-	H	PE	-	15	15	44	-	7.0	22.4	90.9
Chiapasco et al., 2012 [46]	PS	CV	Co	on	RCM/DBBM	V	PTE	-	18	18	60	49.1	5.5	19.0	90.3
	PS	RA	Co	on	RCM/DBBM	V	PTE	-	18	18	60	49.1	5.5	19.0	93.1
Chiapasco et al., 2020 [47]	RS	RA	CoCa	on	RCM/DBBM	HV	PE	Mn	75	75	182	49	-	120	98.1
Cordaro et al., 2002 [48]	PS	io	-	on	-	HV	PE	-	15	15	40	-	6.0	12.0	100
Cordaro et al., 2010 [49]	PS	RA	Co	on	RCM/DBBM	HV	PTE	Mx	16	16	37	51.0	4.0	40.0	100
Cordaro et al., 2011 [50]	RCT	RA	Co	on	-	H	PE	Mn	17	22	27	42.0	4.0	24.0	100
	RCT	RA	Co	on	RCM/DBBM	H	PE	Mn	8	11	28	42.0	4.0	24.0	100
De Santis et al., 2011 [51]	PS	IC	CoCa	in	RCM/DBBM	V	TE	Mx	20	20	154	58.9	4.0	66.4	97.4
De Stavola and Tunkel, 2013 [52]	PS	RA	Co	on	-	V	PE	-	10	10	18	54.0	4.0	12.0	100
Dottore et al., 2013 [53]	PS	io	-	in	-	V	PE	Mn	11	11	22	52.1	6.0	12.0	90.9
Elo et al., 2009 [54]	RS	-	-	on	-	V	PE	-	65	65	184	-	-	36.0	97
Esposito et al., 2015 [55]	RCT	IC	CoCa	on	RCM	HV	TE	Mx	13	13	92	52.0	4.0	12.0	98.9
Felice et al., 2009a [56]	RCT	IC	Ca	on	-	V	PE	Mn	10	10	23	54.0	3.5	18.0	100
	RCT	IC	Ca	in	-	V	PE	Mn	10	10	20	54.0	3.5	18.0	100
Felice et al., 2009b [57]	RCT	IC	Ca	in	-	V	PE	Mn	10	10	19	54.0	4.0	12.0	94.4
Gultekin et al., 2017 [58]	RS	IC	CoCa	on	-	HV	PTE	Mx	18	18	96	48.4	7.5	30.7	96.9
Guo et al., 2020 [59]	RS	RA	Co	in	RCM/DBBM	H	PE	Mn	56	56	72	-	3	36	100
Hartlev et al., 2020 [60]	RCT	RA	-	on	RCM/DBBM	HV	PE	Mx	13	13	13	52.3	6	24	85
	RCT	RA	-	on	PRF	HV	PE	Mx	14	14	14	47.9	6	24	100
Is¸ık et al., 2020 [61]	RCT	RA	CoCa	on	PRF	V	PE	Mn	11	11	24	50.9	6	12	100
Jensen et al., 2006 [62]	RS	RA	Co	in	-	V	PE	Mx	10	10	15	-	4.0	60.0	100
Jemt & Lekholm, 2003 [63]	PS	CH	CoCa	on	-	H	PE	Mx	10	10	10	26.1	6.0	24.0	100
Kablan, 2020 [64]	RS	CV	Co	on	-	HV	PTE	Mn	11	18	63	45	DI	48	100
Kawakami et al., 2013 [65]	PS	RA	Co	in	-	V	PE	Mn	11	11	22	52.1	6.0	12.0	100
Keller et al., 1999 [66]	RS	eo	-	on	-	HV	PTE	Mx	32	32	204	-	5.0	144	86.3
Kim et al., 2013 [67]	RS	RA	Co	on	-	V	PTE	-	28	28	61	43.1	6.2	85.2	94.2
Levin et al., 2007 [68]	RS	io	-	on	-	H	PE	-	50	50	129	45.4	5.2	24.3	96.9
McCarthy et al., 2003 [69]	PS	RA	CoCa	on	-	H	PE	Mx	10	10	35	31.4	-	36.0	97.1
McGrath et al., 1996 [70]	RS	IC	CoCa	on	HA	HV	TE	Mn	18	18	36	57.0	DI	17.0	91.6
Meijndert et al., 2005 [71]	PS	CH	Co	on	-	H	PE	Mx	10	10	10	32.9	3.0	12.0	100
Meijndert et al., 2008 [72]	RCT	io	CoCa	on	-	H	PE	Mn	31	31	31	33.3	3.0	12.0	100
	RCT	io	CoCa	on	RCM	H	PE	Mn	31	31	31	33.3	3.0	12.0	100
	RCT	io	CoCa	on	RCM/DBBM	H	PE	Mn	31	31	31	33.3	3.0	12.0	93.5
Mendoza-Azpur et al., 2019 [73]	RCT	RA	Co	on	RCM/DBBM	H	PE	-	20	20	31	49.6	6.0	18.0	100
Mertens et al., 2012 [74]	RS	IC	-	on	-	V	PTE	-	9	9	34	53.3	6.0	12.3	100
	RS	CV	-	on	-	V	PTE	-	14	14	65	53.3	6.0	12.3	98.5
Molly et al., 2006 [75]	RS	IC	CoCa	on	-	HV	PTE	Mx	18	18	85	45.5	8.0	168	77.2
Nielsen and Jensen, 2020 [76]	RS	RA	CoCa	on	RCM	H	PE	Mn	24	39	48	23	5.5	120	100
Nyström et al., 2004 [77]	PS	IC	CoCa	on	-	HV	TE	Mx	30	30	177	53.2	DI	120	88.7
Nyström et al., 2009 [78]	PS	IC	CoCa	on	-	HV	TE	Mx	44	44	334	58.0	6.0	132	91.9
Pelo et al., 2010 [79]	PS	IC	CoCa	in	-	HV	TE	Mn	19	19	141	58.8	4.0	48.0	93.6
Penarrocha-Diago et al., 2013 [80]	RS	io	-	on	RCM/βTCP	H	PTE	-	21	21	33	48.0	6.8	12.0	96.9
	RS	io	-	on	RCM/βTCP	H	PTE	-	21	24	38	48.0	DI	12.0	100
Penarrocha-Oltra et al., 2014 [81]	RS	RA	CoCa	on	RCM/βTCP	V	PE	Mn	20	20	45	48.4	7.0	12.0	95.6
Pistilli et al., 2014 [23]	RCT	RA/IC	CoCa	on	RCM	HV	PTE	-	20	20	81	49.5	4.0	12.0	98.8
Raghoebar et al., 1996 [82]	PS	io	CoCa	on	-	HV	PE	Mx	27	27	31	36.0	3.0	37.0	100
Raghoebar et al., 2003 [83]	PS	IC	CoCa	on	-	H	TE	Mx	10	10	68	55.5	3.0	12.0	95.6
Sbodorne et al., 2015 [84]	RS	io	Co	on	-	H	PE	-	17	28	73	50.3	4.0	60.0	96.8
Sbordone et al., 2009 [85]	RS	-	-	on	-	HV	PE	-	40	48	48	-	4.0	36.0	99.1
Schwarzt-Arad et al., 2016 [86]	RS	io	CoCa	on	-	HV	PE	-	214	224	667	50.3	5.6	137	93.4
Sethi and Kaus, 2001 [87]	PS	io	Co	on	-	HV	PE	-	60	60	118	47.0	4.5	22.0	98.2
Sjöström et al., 2007 [88]	PS	IC	CoCa	on	-	H	TE	Mx	29	29	222	58.0	6.0	12.0	92.3
Smolka et al., 2006 [89]	PS	CV	Co	on	-	V	TE	Mn	10	10	20	59.3	6.0	30.0	95
Stellingsma et al., 2013 [90]	RCT	IC	CoCa	in	-	HV	TE	Mn	20	20	80	59.4	3.0	76.0	88.8
Stricker et al., 2021 [91]	RS	RA	CoCa	on	-	HV	PE	-	11	16	22	53	3.5	17	100
Thor et al., 2005 [92]	RCT	IC	CoCa	on	-	HV	TE	Mx	19	19	76	58.0	6.0	12.0	97.4
Tosun et al., 2017 [93]	RS	IC	CoCa	on	-	HV	PTE	V	5	5	61	-	3	29	100
	RS	IC	CoCa	on	-	HV	PTE	V	5	5	42	-	DI	29	92.8
Van der Mark et al., 2010 [94]	RS	IC	CoCa	on	-	HV	TE	Mx	17	17	86	53.0	5.5	17.0	97.6
	RS	IC	CoCa	in	-	HV	TE	Mx	10	10	54	53.0	5.5	17.0	94.4
Van der Meij et al., 2005 [95]	RS	IC	CoCa	on	-	V	TE	Mn	17	17	34	56.0	DI	52.0	88.2
Van Steenberghe et al., 1997 [96]	RS	IC	CoCa	-	-	HV	PTE	Mx	13	13	72	49.0	DI	12.0	85
Verhoeven et al., 1997 [97]	PS	IC	CoCa	on	-	V	TE	Mn	13	13	30	59.5	DI	73.0	100
Vermeeren et al., 1996 [98]	PS	IC	CoCa	on	-	V	TE	Mn	31	31	78	51.0	DI	60.0	90
Vinci et al., 2019 [99]	RS	CV	Co	on	-	V	TE	-	32	41	207	61.1	5.0	120	97.1
Widmark et al., 2001 [100]	PS	IC	-	-	-	H	PTE	Mx	20	101	101	-	-	12.0	83.1
Wiltfang et al., 2005 [101]	RS	IC	CoCa	on	-	HV	PTE	Mx	39	39	235	56.3	6.0	54.0	91.5
Wiltfang et al., 2014 [102]	PS	IC	CoCa	on/in	RCM	V	PE	-	40	40	237	58.0	-	24.0	99.2
	PS	IC	CoCa	on/in	RCM/DBBM	V	PE	-	40	40	248	64.7	-	24.0	98.8

**Table 2:** Studies reporting the survival rates of implants placed in allogeneic bone grafts.

Author	SD	GT	Co/Ca	in/on	RB	DT	DS	GL	*n* (P)	*n* (G)	*n* (I)	Age	TtI	F-Up	ISR (%)
Acocella et al., 2012 [103]	PS	FFBA	CoCa	on	-	H	TE	Mx	16	18	34	41.0	6.0	18.0	100
Ahmadi et al., 2017 [104]	PS	FDBA	CoCa	on	RCM	H	PE	Mx	10	10	10	45.0	7.0	15.1	100
Amorfini et al., 2014 [105]	RCT	SDBA	CoCa	on	RCM	HV	PE	Mn	16	16	25	59.5	DI	12.0	100
Aslan et al., 2016 [106]	RS	DFDBA	Co	on	-	H	PTE	-	11	12	32	39.5	5.0	24.0	100
Carinci et al., 2010 [107]	RS	FFBA	CoCa	on	-	HV	PTE	Mx	69	69	287	52.0	5.0	26.0	98.3
Carinci et al., 2009 [108]	RS	FFBA	Co	on	-	HV	TE	Mn	21	28	63	-	6.0	20.0	96.8
Chaushu et al., 2019 [109]	RS	FDBA	Ca	on	RCM/DBBM	HV	PE	Mn	14	14	26	38.0	6.0	26.0	100
Contar et al., 2009 [110]	RS	FFBA	Co	on	-	HV	TE	Mx	15	34	51	44.0	9.0	24.0	100
Deluiz et al., 2016 [111]	PS	FFBA	CoCa	on	-	HV	-	Mx	58	92	268	58.0	5.0	12.0	94.0
Dias et al., 2016 [112]	PS	FFBA	CoCa	on	RCM/DBBM	HV	PE	Mn	12	30	30	50.9	6.0	26.0	96.7
Franco et al., 2009 [113]	RS	FFBA	Co	on	-	HV	PTE	-	36	36	94	53.0	6.0	25.0	95.7
Keith et al., 2006 [114]	PS	SDBA	CoCa	on	RCM	HV	-	-	73	82	97	-	6.0	14.8	99
Maiorana et al., 2016 [115]	PS	FFBA	CoCa	on	RCM	HV	PTE	-	45	45	262	53.9	6.4	26.0	90.8
Chaushu et al., 2009 [116]	PS	FDBA	Ca	on	RCM	HV	PE	Mx	11	12	12	24.0	DI	15.0	100
Nissan et al., 2011a [117]	PS	FDBA	Ca	on	RCM	HV	PE	Mn	21	29	85	55.7	6.0	37.0	95.3
Nissan et al., 2011b [118]	PS	FDBA	Ca	on	RCM	HV	PE	-	12	19	21	21.0	6.0	30.0	95.2
Nissan et al., 2011c [119]	PS	FDBA	Ca	on	RCM	HV	PE	Mx	31	46	63	32.0	6.0	34.0	98
Nissan et al., 2011d [120]	PS	FDBA	Ca	on	RCM	HV	PE	Mx	20	28	31	25.0	6.0	42.0	96.8
Nord et al., 2019 [121]	RS	FDBA Ring	Ca	in	RCM/DBBM	V	PE	-	51	81	81	58.8	DI	12.0	97.5
Novell et al., 2012 [122]	RS	FDBA	CoCa	on	RCM	HV	PTE	-	20	41	62	38.5	6.0	30.7	100
Procopio et al., 2019 [123]	RS	FFBA	CoCa	-	-	HV	TE	-	483	483	1405	51.8	-	60.0	96.2
Silva et al., 2017 [124]	PS	FFBA	CoCa	on	RCM/DBBM	HV	PE	Mn	20	50	50	51.8	6.0	31.8	96
Tresguerres et al., 2019 [125]	RCT	FDBA	CoCa	on	PRGF	H	PTE	Mx	28	14	53	65.8	4.0	24.0	100
	RCT	FDBA	Co	on	PRGF	H	PTE	Mx	28	14	39	65.8	4.0	24.0	100

**Table 3:** Comparative studies reporting the survival rates of implants placed in autogenous and allogeneic bone grafts.

Author	SD	GT	Co/Ca	in/on	RB	DT	DS	GL	*n* (P)	*n* (G)	*n* (I)	Age	TtI	F-Up	ISR (%)
Al-Abedalla et al., 2015 [126]	PS	-	CoCa	on	-	-	PE	-	43	43	83	57.8	5.5	34.8	96.4
	PS	PBA	CoCa	on	-	-	PE	-	16	16	63	57.8	5.7	31.1	96.8
Chiapasco et al., 2015 [127]	PS	IC	CoCa	on	-	HV	TE	Mx	7	7	49	56.0	4.5	24.0	100
	PS	FFBA	CoCa	on	-	HV	TE	Mx	8	8	59	56.0	4.5	24.0	90.1
Dellavia et al., 2016 [128]	PS	IC	CoCa	on	RCM	-	PTE	-	6	6	32	53.0	8.0	15.0	100
	PS	FFBA	CoCa	on	RCM	-	PTE	-	14	14	69	53.0	7.0	15.0	96.8
Kloss et al., 2018 [129]	RS	RA	CoCa	on	RCM	HV	PE	-	21	21	21	48.0	6.0	12.0	100
	RS	FDBA	Ca	on	RCM	H	PE	-	21	21	21	48.0	6.0	12.0	100
Park et al., 2017 [130]	RS	io	Co	on	RCM	V	PE	-	9	9	15	53.0	5.1	32.9	100
	RS	FDBA	CoCa	on	RCM	V	PE	-	12	12	26	53.0	7.5	32.9	100
Schlee et al., 2014 [16]	RS	RA	-	on	RCM	HV	PE	-	21	21	33	49.3	6.0	59.5	100
	RS	SDBA	Ca	on	RCM	HV	PE	-	10	10	15	49.3	6.0	28.6	100

**Table 4:** Overview of patients (*n*(P)), grafts (*n*(G)) and implants (*n*(I)) included into the analysis for respective subgroups. Mean ISR (M), standard deviation (SD), and p-values from rank-analysis of the overall ISR with all established sub-entities via the Kruskal–Wallis test ^†^ and multiple comparisons of the overall ISRs with each sub-entity via the uncontrolled Dunn’s test ^‡^ (*p ≤ 0.05*).

Implant Survival Rate (%)						
Autograft	*n* (P)	*n* (G)	*n* (I)	M	SD	*p*
1. Overall	2195	2397	6861	96.23	5.19	<0.0001 ***^,^^†^
2a. Intraoral	1248	1327	2557	97.97	3.30	0.045 *^,^^‡^
2b. Ramus	484	534	982	98.46	3.42	0.011 *^,^^‡^
2c. Extraoral	708	805	3797	93.88	6.32	0.023 *^,^^‡^
2d. Iliac crest	591	672	3178	93.76	6.56	0.035 *^,^^‡^
3a. Cortical	431	488	1111	98.39	2.75	0.048 *^,^^‡^
3b. Cortico-cancellous	1185	1215	4469	95.24	5.75	0.21 ^‡^
4a. Onlay graft	1914	1995	5929	96.45	5.06	0.89 ^‡^
4b. Inlay graft	217	239	728	96.65	3.88	0.83 ^‡^
5a. Resorption barrier	821	872	2052	97.58	3.69	0.24 ^‡^
5b. Collagen membrane	257	299	761	99.26	1.72	0.021 *^,^^‡^
5c. Relining	509	518	1186	96.54	4.14	0.84 ^‡^
5d. Without resorption barrier	1343	1476	4778	95.38	5.76	0.35 ^‡^
6a. Horizontal augmentation	617	740	1439	96.91	4.31	0.60 ^‡^
6b. Vertical augmentation	1498	1559	5276	95.84	5.55	0.66 ^‡^
7a. Partially edentulous	1345	1426	3175	98.08	3.13	0.049 *^,^^‡^
7b. Totally edentulous	430	439	2180	93.92	5.70	0.021 *^,^^‡^
8a. Maxilla	496	577	2407	93.75	7.27	0.16 ^‡^
8b. Mandible	558	610	1355	96.86	4.05	0.54 ^‡^
9a. Young patients	211	234	342	98.61	2.41	0.078 ^‡^
9b. Old patients	1581	1651	5436	96.18	4.71	0.61 ^‡^
11a. Immediate implantation	234	246	735	91.56	7.22	0.023 *^,^^‡^
11b. Early implantation	1076	1164	3276	97.60	3.40	0.22 ^‡^
11c. Late implantation	818	831	2499	95.93	5.23	0.75 ^‡^
12a. Short follow-up	900	1024	2853	96.55	5.37	0.51 ^‡^
12b. Long follow-up	1264	1324	3977	95.70	5.00	0.35 ^‡^
Allograft	n (P)	n (G)	n (I)	M	SD	p
1. Overall	1202	1384	3434	97.66	2.68	0.342 ^†^
2a. Fresh-frozen BA	797	907	2672	95.94	2.95	0.062 ^‡^
2b. Processed BA	405	477	762	98.81	1.70	0.15 ^‡^
3a. Cortical	111	124	279	98.50	1.87	0.53 ^‡^
3b. Cancellous	191	260	355	98.08	1.92	0.82 ^‡^
3c. Cortico-cancellous	855	955	2538	97.59	2.74	0.89 ^‡^
4. Onlay graft	668	820	1948	97.72	2.76	0.88 ^‡^
5a. Resorption barrier	413	560	986	97.89	2.46	0.86 ^‡^
5b. Collagen membrane	316	385	799	97.99	2.66	0.67 ^‡^
5c. Relining *	97	175	187	97.54	1.52	0.65 ^‡^
5d. without resorption barrier	789	824	2448	97.33	2.95	0.82 ^‡^
6a. Horizontal augmentation	114	89	189	100.00	0.00	0.022 *^,^^‡^
6b. Vertical augmentation	1058	1265	3113	97.11	2.81	0.41 ^‡^
7a. Partially edentulous	277	394	559	98.14	1.87	0.73 ^‡^
7b. Totally edentulous	543	571	1612	96.62	3.62	0.68 ^‡^
8a. Maxilla	294	345	907	97.93	3.07	0.53 ^‡^
8b. Mandible	104	167	279	97.44	1.88	0.58 ^‡^
9a. Early implantation	218	225	801	97.03	3.49	0.85 ^‡^
9b. Late implantation	423	567	1110	97.73	2.45	0.94 ^‡^
10a. Young patients	150	224	332	98.89	1.71	0.21 ^‡^
10b. Old patients	958	1050	2942	97.06	2.96	0.47 ^‡^
11a. Short follow-up	381	456	914	98.28	2.80	0.31 ^‡^
11b. Long follow-up	821	928	2520	96.00	2.40	0.31 ^‡^

^†^ Kruskal–Wallis test; ^‡^ uncontrolled Dunn’s test. (*p <* 0.05 */0.0005 ***)

**Table 5:** Statistical analysis of nonparametric data with Kruskal–Wallis test for multiple comparisons and Mann–Whitney U test for pairwise comparisons (*p <* 0.05). A, AUBB, B = ALBB; Co.-Ca., cortico-cancellous; w/o RB, without resorption barrier; part. Edent., partially edentulous; imme., immediate; FUP, follow-up. (*p <* 0.05 */0.005 **/0.0005 ***).

Overall (A)	Overall (B)	Overall (A)		Processed BA (B)		Fresh-Frozen BA (B)	Overall (B)	Intraoral (A)	Extraoral (A)
vs.	0.54	vs.		0.07		0.98	vs.	0.94	0.002 **
				vs.		0.004 **		vs.	0.0003 ***
**Ramus (A)**	**Ilium (A)**	**Co.-Ca. (A)**	**Cortical (A)**	**Co.-Ca. (B)**	**Cortical (B)**	**Cancellous (B)**	**Onlay (A)**	**Inlay (A)**	**Onlay (B)**
vs.	0.0002 ***	vs.	0.005 **	0.23	*-*	*-*	vs.	0.90	0.55
			vs.	-	0.89	-			
				vs.	>0.99	>0.99			
**Res.-Barrier (A)**	**w/o RB (A)**	**Res.-Barrier (B)**		**w/o RB (B)**		**Membrane (A)**	**Relining (A)**	**Membrane (B)**	Relining * (B)
vs.	0.071	0.74		-		vs.	0.019 *	0.21	-
	vs.	-		0.74			vs.	-	0.97
		vs.		0.48				vs.	0.50
**Horizontal (A)**	**Vertical (A)**	**Horizontal (B)**		**Vertical (B)**		**Part. Edent. (A)**	**Totally Edent. (A)**	**Part. Edent. (B)**	**Totally Edent. (B)**
vs.	0.44	0.053		-		vs.	0.0002 ***	0.53	-
	vs.	-		0.091			vs.	-	0.33
		vs.		0.009 **				vs.	0.57
**Maxilla (A)**	**Mandible (A)**	**Maxilla (B)**		**Mandible (B)**		**<45 Years (A)**	**>45 Years (A)**	**<45 Years (B)**	**>45 Years (B)**
vs.	0.11	0.10		-		vs.	0.044 *	>0.99	-
	vs.	-		0.96			vs.	-	0.87
		vs.		0.40				vs.	0.11
**Imme. Implantation (A)**	**<6 mon. (A)**	**≥6 mon. (A)**		**<6 mon. (B)**	**≥6 mon. (B)**	**Short-FUP (A)**	**Long-FUP (A)**	**S-FUP (B)**	**L-FUP (B)**
vs.	0.018 *	0.21		vs.	0.90	vs.	0.17	0.35	-
	vs.	0.66		0.66	-		vs.	-	0.86
		vs.		*-*	0.48			vs.	0.082

**Figure A1:**
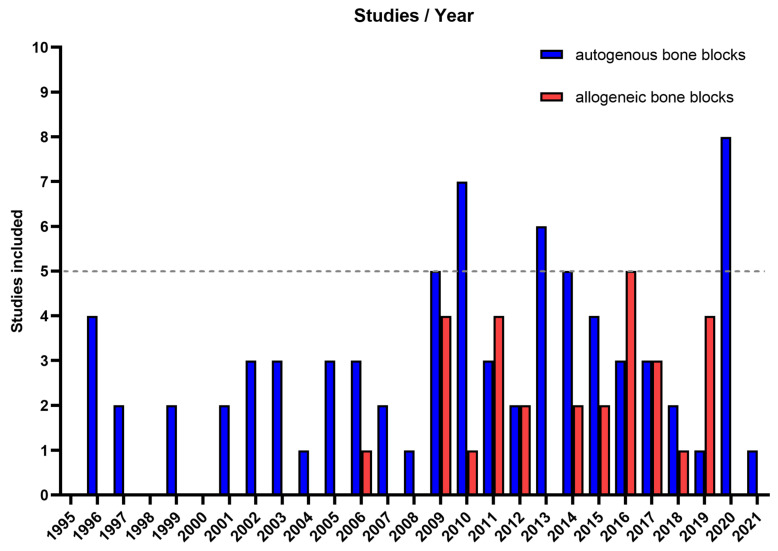
Distribution of the included studies by year of publication.

## Data Availability

Primary data including calculations will be provided by the corresponding author upon reasonable request.

## Appendix B

**Table A1:** List of excluded studies with reasons for exclusion.

Study	Reason for Exclusion
Aghazadeh et al., 2012 [140]	Application of granular autograft
Arenaz-Búa et al., 2010 [141]	Application of granular autograft
Barboza et al., 2010 [142]	Application of allogeneic bone granules
Beitlitum et al., 2018 [143]	Application of allogeneic bone granules
Bianconi et al., 2017 [144]	Alveolar ridge preservation
Charde et al., 2020 [145]	Block used for peri-implant bone regeneration
Naishlos et al., 2021 [146]	Blocks used for sinus floor augmentation
Corinaldesi et al., 2009 [147]	Application of autogenous bone granules
El Chaar et al., 2019 [148]	Application of allogeneic bone granules
Ge et al., 2017 [149]	Application of granular autograft
Güven and Tekin, 2006 [150]	Wrong indication (cyst filling)
Huang et al., 2016 [151]	Application of allogeneic bone granules
Ilankovan et al., 1998 [152]	No outcome of interest reported
Jacotti et al., 2012 [153]	Sample size too small
Kang et al., 2015 [154]	Particulated iliac bone applied with sinus lift
Khoury and Hanser, 2015 [155]	No outcome of interest reported
Krasny et al., 2018 [156]	Inclusion of patients with follow-up of less than 12 months
Lekholm et al., 1999 [157]	Application of different surgical approaches, missing patient information
Lima et al., 2018 [158]	Sample size too small
Margonar et al., 2010 [159]	Combination of autogenous and allogeneic bone
Mau et al., 2019 [160]	Application of granular materials
Merli et al., 2020 [161]	Application of granular grafting materials
Morad and Khojasteh, 2013 [162]	Sample size too small
Mordenfeld et al., 2017 [163]	Application of granular autograft
Özkan et al., 2007 [164]	Sample size too small
Pimentel et al., 2014 [165]	Sample size too small
Putters et al., 2018 [166]	No outcome of interest reported
Quereshy et al., 2010 [167]	Sample size too small
Sethi et al., 2020 [168]	Inclusion of patients with follow-up < 12 months
Simion et al., 2001 [169]	Application of granular materials
Simon et al., 2000 [170]	Surgical technique (ARP)
Soehardi et al., 2009 [171]	No differentiation between onlay block and sinus floor elevation
Solakoglu et al., 2019 [172]	Application of allogeneic bone granules
Stellingsma et al., 2003 [173]	No outcome of interest reported
Tresguerres et al., 2021 [174]	Inclusion of cancer and metabolic-disease patients
Zou et al., 2015 [175]	Treatment of cancer patients

## Appendix C. Modified National Institute of Health Quality Assessment Tool (mNIH)

The National Institutes of Health (NIH) Study Quality Assessment Tool for Case Series Studies (https://www.nhlbi.nih.gov/health-topics/study-quality-assessment-tools; accessed on 5 August 2021) was used for assessing the quality of all studies included in the systematic review. The original tool is composed of nine quality-related questions which were adapted by evaluation of the study type, the presence of a registered study protocol and adequacy of the sample size. Furthermore, split points were assigned to the study design, in which RCTs received a score of 1, PSs received a score of 0.5, and RSs and CSs received a score of 0. The item “registered study protocol” was fulfilled when a protocol number was provided or the authors had a striking affiliation with a university clinic and mentioned an existing protocol; however, studies solely stating that the “study protocol was registered” without any further information were attributed 0 points. If studies were missing information regarding their objective, 0.5 points were deducted. Regarding the description of the study population, 0.5 points were deducted when a description of patient age, treated sites, dental status or follow-up times were not fully described, whereby 0 points were given if multiple information points were missing. This was conducted similarly for the comparability of study populations; if the age of the oldest patient was more than twice as high as that of the youngest participant, 0.5 points were deducted. If further striking differences regarding treatment schedules or patient-related factors were found, no points were assigned. If studies reported consecutiveness, they were attributed one point. Depending on the sample size of <20, 20–30 and >30 participants, studies were awarded 0, 0.5 and 1 point, respectively. Clear descriptions of the intervention and outcome measures included precise descriptions of applied materials, surgical techniques and measuring methods together with their chronological conduction. Depending on the amount of information missing, 0, 0.5 or 1 points were assigned for both criteria, respectively. Adequacy of the length of follow-up was set to 1 point for studies reporting more than 36 months, 0.5 for those reporting 25 to 36 months, and 0 for those reporting 24 months or less. Statistical data descriptions were checked for completion and the possibility of reproduction; if information was lacking or the statistical software was not mentioned, 0.5 points were deducted. In cases of an absence of statistical analyses and solely descriptive data being provided, studies were excluded from this item. If results were insufficiently described, no or redundant figures were presented or the data illustration was inconsistent, 0.5 points were deducted, in cases where more criteria for point deductions were fulfilled, no points were assigned. A listing with a color coding of study scoring, in which green resembles 1 point, yellow 0.5 points and red 0 points, was elaborated and is depicted below with an exemplary illustration of the evaluation scheme.

**Table A2:** Modified NIH Quality Assessment Tool for the case series.

Item	1	0.5	0	Ex.
**1. Which study design was chosen?**				
**2. Was there a registered protocol?**				
**3. Was the study question or objective clearly stated?**				
**4. Was the study population clearly and fully described, including a case definition?**				
**5. Were the cases consecutive?**				
**6. Were the subjects comparable?**				
**7. Was the sample size robust (10–19/20–29/30-X)?**				
**8. Was the intervention clearly described?**				
**9. Were the outcome measures clearly defined, valid, reliable and implemented consistently across all study participants?**				
**10. Was the length of follow-up adequate? (12–23/24–35/36-X)**				
**11. Were the statistical methods well-described?**				
**12. Were the results well-described?**				

**Table A3:** Evaluation sheet for the mNIH scoring tool with color coding (green = 1 point, yellow = 0.5 points, red = 0 points, blue = item not applicable).

Author	1	2	3	4	5	6	7	8	9	10	11	12	Score
Al-Abedalla et al., 2015	PS												0.83
Amorfini et al., 2014	RCT												0.92

## Appendix D. Newcastle–Ottawa Scale

Non-randomized cohort studies were evaluated with the Newcastle-Ottawa Scale (NOS), which includes eight quality-associated items in three areas of interest regarding study selection, group comparability and the ascertainment of exposure and outcome [31]. A great consistency in the evaluation item design between the NIH and NOS exists; however, only 1 or 0 points were assigned according to the NOS protocol, so the color coding was either red or green and the final result was divided by eight to obtain the score.

**Table A4:** Newcastle–Ottawa Scale for the assessment of cohort studies.

Item	Maximum Points
**1. Representativeness of exposed cohort**	1
**2. Selection of external control**	1
**3. Ascertainment of exposure**	1
**4. Outcome of interest is not present at the start**	1
**5. Comparability of cohorts on the basis of the design or analysis**	1
**5a. Study controls for an additional factor**	1
**6. Assessment of outcome**	1
**7. Was follow-up long enough for outcome to appear?**	1
**8. Adequate documentation about follow-up of cohorts**	1

**Table A5:** Evaluation sheet for the NOS with color coding (green = 1 point, red = 0 points). Overall result of points divided by the number of evaluated items resulted in the quality score.

Author	1	2	3	4	5	5a	6	7	8	Score
Al-Abedalla et al., 2015										1.00
Beitlitum et al., 2010										0.75

## Appendix E. Jadad Scale

The quality of randomized controlled trials was assessed by the Jadad scale, which is a simple scoring tool to assess the methodologic quality of clinical trials by three criteria: randomization, blinding and patient follow-up and documentation [32]. The overall achievable score using the Jadad scale is 5 points, if randomization and blinding as well as the fate of all study subjects are mentioned and described, although one point is being deducted if randomization or blinding are inappropriately described or not mentioned; therefore, the following color coding for the evaluation of studies was applied: green = 1 point, yellow = 0 points, and red = −1 point. An exemplary scheme is displayed below.

**Table A6:** Evaluation sheet for the Jadad scale with color coding (green = 1 point, yellow = 0 points, red = −1 point). Overall result of points divided by the number of evaluated items resulted in the score.

Author	1	2	3	4	5	6	7	Score
Amorfini et al., 2014								1.00
Avila-Ortiz et al., 2020								0.80

**Table A7:** Jadad scale for quality assessment of controlled trials.

Item	Description	Maximum Points
**1. Randomization**	Randomization is mentioned	1
	Appropriate randomization method	1
	Inappropriate randomization method	−1
**2. Blinding**	Blinding is mentioned	1
	Appropriate blinding method	1
	Inappropriate blinding method	−1
**3. Account of all patients**	Fate of all patients in the trial is known and reasoned otherwise	1

## Appendix F

**Table A8:** Quality assessment of studies on AUBB via mNIH.

Author	1	2	3	4	5	6	7	8	9	10	11	12	mNIH
Acocella et al., 2010	RS												0.46
Altiparmak et al., 2020	RS												0.79
Astrand et al., 1996	RS												0.50
Bartols et al., 2018	RCT												0.92
Bell et al., 2002	RS												0.67
Bienz et al., 2020	RCT												0.92
Bormann et al., 2010	PS												0.73
Bormann et al., 2011	RS												0.73
Boronat et al., 2010	RS												0.59
Boven et al., 2014	RS												0.83
Buser et al., 2002	PS												0.83
Chappius et al., 2016	PS												0.75
Chiapasco et al., 1999	PS												0.53
Chiapasco et al., 2012	PS												0.59
Chiapasco et al., 2020	RS												0.71
Cordaro et al., 2002	PS												0.59
Cordaro et al., 2010	PS												0.63
Cordaro et al., 2011	RCT												0.79
De Santis et al., 2011	PS												0.79
De Stavola and Tunkel, 2013	PS												0.73
Dottore et al., 2014	PS												0.50
Elo et al., 2009	RS												0.67
Esposito et al., 2015	RCT												0.67
Felice et al., 2009a	RCT												0.63
Felice et al., 2009b	RCT												0.79
Gultekin et al., 2017	RS												0.75
Guo et al., 2020	RS												0.75
Hartlev et al., 2020	RCT												0.88
Is¸ık et al., 2020	RCT												0.73
Jemt and Lekholm, 2003	PS												0.73
Jensen et al., 2006	RS												0.55
Kablan, 2020	RS												0.50
Kawakami et al., 2014	PS												0.67
Keller et al., 1999	RS												0.83
Kim et al., 2013	RS												0.79
Levin et al., 2007	RS												0.67
McCarthy et al., 2003	PS												0.54
McGrath et al., 1996	RS												0.36
Meijndert et al., 2005	PS												0.55
Meijndert et al., 2008	RCT												0.88
Mendoza-Azpur et al., 2019	RCT												0.83
Mertens et al., 2012	RS												0.88
Molly et al., 2006	RS												0.75
Nielsen and Jensen, 2020	RS												0.79
Nyström et al., 2004	PS												0.83
Nyström et al., 2009	PS												0.83
Pelo et al., 2010	PS												0.63
Penarrocha-Diago et al., 2013	RS												0.67
Penarrocha-Oltra et al., 2014	RS												0.71
Pistilli et al., 2014	RCT												0.79
Raghoebar et al., 1996	PS												0.59
Raghoebar et al., 2003	PS												0.68
Sbordone et al., 2009	RS												0.79
Sbordorne et al., 2015	RS												0.63
Schwartz-Arad et al., 2016	RS												0.71
Sethi and Kaus, 2001	PS												0.67
Sjöström et al., 2007	PS												0.79
Smolka et al., 2006	PS												0.50
Stellingsma et al., 2014	RCT												0.83
Stricker et al., 2021	RS												0.63
Thor et al., 2005	RCT												0.79
Tosun et al., 2017	RS												0.54
Van der Mark et al., 2011	RS												0.63
Van der Meij et al., 2005	RS												0.64
Van Steenberghe et al., 1997	RS												0.63
Verhoeven et al., 1997	PS												0.58
Vermeeren et al., 1996	PS												0.67
Vinci et al., 2019	RS												0.77
Widmark et al., 2001	PS												0.55
Wiltfang et al., 2005	RS												0.71
Wiltfang et al., 2014	PS												0.79
**0.74**													

**Table A9:** Quality assessment of studies on AUBB via NOS.

Author	1	2	3	4	5	5a	6	7	8	NOS
Altiparmak et al., 2020										0.75
Chiapasco et al., 1999										0.75
Elo et al., 2009										0.50
Gultekin et al., 2017										0.88
Kawakami et al., 2014										1.00
Keller et al., 1999										1.00
Meijndert et al., 2005										0.88
Molly et al., 2006										1.00
Monje et al., 2015										0.88
Penarrocha-Diago et al., 2013										0.75
Penarrocha-Oltra et al., 2014										0.75
Sbordone et al., 2009										0.75
Sbordorne et al., 2015										0.88
Tosun et al., 2017										0.88
Van der Mark et al., 2011										0.75
Widmark et al., 2001										0.88
Wiltfang et al., 2005										0.75
Wiltfang et al., 2014										0.88
**0.83**										

**Table A10:** Quality assessment of studies on AUBB via Jadad.

Author	1	2	3	4	5	6	7	Jadad
Bartols et al., 2018								0.80
Bienz et al., 2020								0.20
Cordaro et al., 2011								0.40
Esposito et al., 2015								1.00
Felice et al., 2009a								0.60
Felice et al., 2009b								1.00
Hartlev et al., 2020								0.80
Isik et al., 2020								0.80
Meijndert et al., 2008								0.20
Mendez et al., 2017								0.40
Mendoza-Azpur et al., 2019								1.00
Pistilli et al., 2014								1.00
Stellingsma et al., 2014								0.20
Thor et al., 2005								0.20
**0.61**								

## Appendix G

**Table A11:** Quality assessment of studies on ALBB via mNIH.

Author	1	2	3	4	5	6	7	8	9	10	11	12	mNIH
Acocella et al., 2012	PS												0.63
Ahmadi et al., 2017	PS												0.63
Amorfini et al., 2014	RCT												0.83
Aslan et al., 2016	PS												0.79
Carinci et al., 2009	RS												0.67
Carinci et al., 2010	RS												0.59
Chaushu et al., 2019	PS												0.83
Contar et al., 2009	RS												0.50
Deluiz et al., 2016	PS												0.88
Dias et al., 2016	PS												0.83
Franco et al., 2009	RS												0.75
Keith et al., 2006	PS												0.64
Maiorana et al., 2016	RS												0.73
Chaushu et al., 2009	PS												0.63
Nissan et al., 2011a	PS												0.75
Nissan et al., 2011b	PS												0.79
Nissan et al., 2011c	PS												0.82
Nissan et al., 2011d	PS												0.88
Nord et al., 2019	RS												0.75
Novell et al., 2012	RS												0.63
Procopio et al., 2019	RS												0.71
Silva et al., 2017	PS												0.88
Tresguerres et al., 2019	RCT												0.83
**0.80**													

**Table A12:** Quality assessment of studies on ALBB via NOS.

Author	1	2	3	4	5	5a	6	7	8	NOS
Keith et al., 2006										1.00
**1.00**										

**Table A13:** Quality assessment of studies on ALBB via NOS.

Author	1	2	3	4	5	6	7	Jadad
Amorfini et al., 2014								1.00
Tresguerres et al., 2019								0.40
**0.70**								

## Appendix H

**Table A14:** Quality assessment of studies on AUBB and ALBB via mNIH.

Author	1	2	3	4	5	6	7	8	9	10	11	12	mNIH
Al-Abedalla et al., 2015	PS												0.79
Chiapasco et al., 2015	PS												0.71
Dellavia et al., 2016	PS												0.60
Kloss et al., 2018	RS												0.63
Park et al., 2017	RS												0.71
Schlee et al., 2014	RS												0.67
**0.69**													

**Table A15:** Quality assessment of studies on AUBB and ALBB via NOS.

Author	1	2	3	4	5	5a	6	7	8	NOS
Al-Abedalla et al., 2015										1.00
Chiapasco et al., 2015										1.00
Dellavia et al., 2016										0.63
Kloss et al., 2018										1.00
Park et al., 2017										1.00
Schlee et al., 2014										0.88
**0.92**										
